# MBE Growth of High-Quality HgCdSe for Infrared Detector Applications

**DOI:** 10.3390/ma18153676

**Published:** 2025-08-05

**Authors:** Zekai Zhang, Wenwu Pan, Gilberto A. Umana Membreno, Shuo Ma, Lorenzo Faraone, Wen Lei

**Affiliations:** Department of Electrical, Electronic and Computer Engineering, The University of Western Australia, Perth, WA 6009, Australia; zekai.zhang@uwa.edu.au (Z.Z.); wenwu.pan@uwa.edu.au (W.P.); gilberto.umanamembreno@uwa.edu.au (G.A.U.M.); shuo.ma@research.uwa.edu.au (S.M.); lorenzo.faraone@uwa.edu.au (L.F.)

**Keywords:** HgCdSe, infrared detector, molecular beam epitaxy (MBE), mid-wavelength infrared (MWIR)

## Abstract

HgCdSe has recently been proposed as a potential alternative material to HgCdTe for fabricating high-performance infrared detectors. This work presents a study on the growth of high-crystalline-quality HgCdSe materials on GaSb (211)B substrates via molecular beam epitaxy and demonstration of the first prototype HgCdSe-based mid-wave infrared detectors. By optimizing the MBE growth parameters, and especially the thermal cleaning process of the GaSb substrate surface prior to epitaxial growth, high-quality HgCdSe material was achieved with a record XRD full width at half maximum of ~65 arcsec. At a temperature of 77 K, the mid-wave infrared HgCdSe n-type material demonstrated a minority carrier lifetime of ~1.19 µs, background electron concentration of ~2.2 × 10^17^ cm^−3^, and electron mobility of ~1.6 × 10^4^ cm^2^/Vs. The fabricated mid-wave infrared HgCdSe photoconductor presented a cut-off wavelength of 4.2 µm, a peak responsivity of ~40 V/W, and a peak detectivity of ~1.2 × 10^9^ cmHz^1/2^/W at 77 K. Due to the relatively high background electron concentration, the detector performance is lower than that of state-of-the-art low-doped HgCdTe counterparts. However, these preliminary results indicate the great potential of HgCdSe materials for achieving next-generation IR detectors on large-area substrates with features of lower cost and larger array format size.

## 1. Introduction

Infrared (IR) detectors and related sensing and imaging technologies have been widely applied in various industry sectors such as remote sensing, night vision, environmental monitoring, and many others. For all these applications, the most critical electronic components are the high-performance IR detectors and focal plane arrays. Among the various IR technologies currently available in the market, HgCdTe-based IR detectors have dominated the high-performance end of the market for decades due to their superior performance and characteristics such as low dark current, high quantum efficiency, high detectivity, and others. However, high-performance HgCdTe-based IR detectors are typically grown on lattice-matched Cd_0.96_Zn_0.04_Te substrates which have severe limitations due to their relatively lower crystal quality, small wafer size, and high unit price relative to other semiconductor substrates. This leads to a lower yield, higher cost, and smaller array format size for HgCdTe IR detectors based on CdZnTe substrates. For next-generation IR detectors with features of lower cost and larger array format size [1], significant research effort has been undertaken towards growing high-quality HgCdTe materials on alternative substrates such as Si, Ge, GaAs, and GaSb. However, the quality of HgCdTe materials grown on these alternative substrates is still not comparable to that grown on lattice-matched Cd_0.96_Zn_0.04_Te substrates due to the large lattice mismatch between HgCdTe and these alternative substrates [2,3,4]. Along with the effort to grow HgCdTe on alternative substrates, HgCdSe has emerged as a promising alternative approach to address these challenges. With comparable IR capability to HgCdTe [5,6,7], HgCdSe is nearly lattice-matched to GaSb, which is a mature III-V substrate technology with features of low cost, large wafer size, and epi-ready availability. These features render HgCdSe a compelling candidate for achieving next-generation IR detectors with the features of lower cost and larger array format size. Furthermore, since HgCdSe belongs to the family of materials with a lattice constant of around 6.1 Å, including ZnTe, InAs, and AlSb, it provides the potential for monolithic integration of multicolour detectors covering from ultraviolet (UV) to IR wavelengths [2,3,8].

Since the first report of the IR capability of HgCdSe [9], efforts have been undertaken in the realm of MBE growth of HgCdSe materials on GaSb substrates [5,10,11]. While preliminary characterizations have indicated comparable IR physical properties between HgCdSe and HgCdTe in terms of electrical, structural, optical, and mechanical properties [10,12], the reported crystalline quality of Hg_1−x_Cd_x_Se materials typically have a full width at half maximum (FWHM) of the X-ray diffraction (XRD) peaks from 120 to 220 arcsec, which is inferior to HgCdTe grown on Cd_0.96_Zn_0.04_Te substrates (typically ≤ 60 arcsec) [1]. This lower crystalline quality of HgCdSe materials has led to a reluctance to fabricate HgCdSe IR detectors, which needs to be well understood and addressed. In this work, we report on the MBE growth of high-quality mid-wave infrared (MWIR) HgCdSe layers on GaSb (211)B substrates and the first demonstration of a prototype MWIR photoconductive detector based on these MBE-grown HgCdSe materials. By comprehensively optimizing the substrate preparation processes and adopting the optimum growth parameters including flux ratio, beam equivalent pressure (BEP), and growth temperatures obtained from previous research, the HgCdSe epi-layers grown on GaSb substrates in this work present superior crystal quality (XRD FWHM ~ 65 arcsec), which is comparable to that of Hg_1−x_Cd_x_Te grown on Cd_0.96_Zn_0.04_Te. Since the first published MBE-growth of HgCdSe epi-layers in the early 1990s [11], this is the first fabricated IR detector based on MBE-growth of HgCdSe epi-layer, to the best of our knowledge. The fabricated MWIR detectors demonstrate a cut-off wavelength of ~4.2 µm, a peak responsivity of ~40 V/W (at 4 µm), and a peak specific detectivity (D_λ_*) of ~1.2 × 10^9^ cmHz^1/2^/W (at 4 µm) at 77 K.

## 2. Experimental Details

In this work, the MBE growth of HgCdSe materials on GaSb (211)B substrates was undertaken in a Riber 32P system with Hg (7N), Cd (6N), Se (5N5), Zn (6N), and Te (6N) as source materials. The GaSb (211)B substrates were purchased from Wafer Technology, Ltd. with a carrier concentration in the range of 1~9 × 10^17^ cm^−3^. The (211)-orientated crystal orientation was chose for the substrates due to the fact that Hg atoms present a higher sticking coefficient on the (211)-orientated surface [2]. Prior to the epitaxial growth, GaSb (211)B substrates were first thermally cleaned in the MBE chamber at 510 °C for 15 min to remove the native oxide layer on the surface. Subsequently, the substrate temperature was reduced to 320 °C for growth of a ZnTe buffer layer (~200 nm), which serves to electrically isolate the GaSb substrate from the HgCdSe active epi-layer and to block any potential elemental diffusion from the III-V substrate into the II-VI active layer [13]. After growth of the ZnTe buffer layer, the substrate temperature was reduced to 85 °C for growth of the HgCdSe layer (~5 µm). Because of the low sticking coefficient of Hg, a large Hg beam equivalent pressure (BEP) of ~10^−4^ Torr was used for growing HgCdSe, while relatively lower BEPs were used for Se (~10^−6^ Torr) and Cd (~10^−7^ Torr). The MBE growth was evaluated with in situ tools such as reflection high-energy electron diffraction (RHEED), and post-growth tools such as X-ray diffraction (XRD). Fourier transform infrared (FTIR) spectroscopy was used to evaluate the optical properties of the HgCdSe epi-layers including cut-off wavelength and optical absorption/transmission. Van der Pauw Hall measurements combined with high-resolution mobility spectrum analysis (HR-MSA) was used to study the carrier transport properties of HgCdSe epi-layers, which can readily separate the carrier transport contribution from the HgCdSe epi-layer from that of the conductive GaSb substrate. Photoconductive decay measurements under low-level carrier injection conditions were undertaken to study the minority carrier lifetime in the HgCdSe epi-layers.

**Figure 1:**
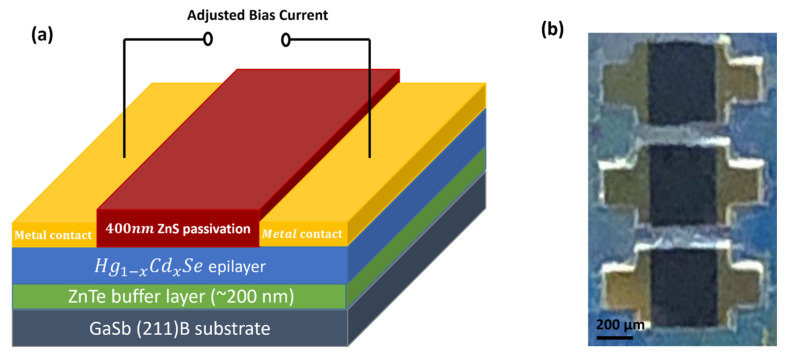
(**a**) Schematic device structure of HgCdSe photoconductor in this work; (**b**) optical microscope image of HgCdSe photoconductors fabricated in this work.

Figure 1 shows the structure of the HgCdSe photoconductors fabricated in this work. Standard photolithographic processes were used to define the detector mesa dimensions (square shape with a dimension of 600 µm × 600 µm). Subsequently, a wet etching process using a Br_2_/HBr (0.5%) solution was employed to define the detector mesa structures. Then, bilayers of Cr/Au (20 nm/200 nm) were thermally evaporated to form metal contacts, and a 400 nm thick ZnS layer was thermally evaporated onto the devices to serve as a passivation layer. The devices were then manually bonded to external contacts using E-solder 3021 silver epoxy (Von Roll, Inc., New Haven, CT, USA) and gold wires. Responsivity measurements on the HgCdSe photoconductors were performed using an Optronic Laboratories Detector Spectral Response Measurement System which consists of a 750-20 dual source attachment, a 750-D monochromator, and a 750-C automated Spectroradiometer system (Gooch & Housego PLC, Ilminster, UK). Noise spectrum measurements were conducted using a Hewlett Packard 35665A dynamic signal analyser (Keysight, Santa Rosa, CA, USA), and dark current analysis was performed with a Keithley 4200 SCS semiconductor parameter analyser (Tektronix, Inc., Beaverton, OR, USA).

## 3. Results and Discussion

### 3.1. Influence of Thermal Cleaning on the HgCdSe Crystalline Quality

For MBE growth of thin film materials, one of the important growth steps is the thermal cleaning/desorption of the substrate surface. The strong correlation between the substrate condition and the quality of the subsequently grown thin film has been widely reported [14,15] and the ideal substrate surface prior to epitaxial growth should be mirror-like, smooth, and free of any oxides [16]. Since GaSb substrates are reported to rapidly oxidize in ambient atmosphere [17], it is critical to achieve complete thermal cleaning/desorption of any native oxide from the GaSb substrate surface and to create a clean, smooth growth front for the subsequent growth of the epi-layers. Although the MBE growth parameters for HgCdSe and ZnTe such as flux ratio and growth temperature have been comprehensively studied, there is a lack of information in the open literature regarding optimization of the thermal cleaning process of GaSb substrates, which could be the reason for the relatively large values of XRD FWHM for HgCdSe and needs to be well understood and addressed.

It has been reported that an oxide-free surface of GaSb can be achieved at temperatures between 500 °C and 550 °C [15,17,18]. Schwartz et al. [19] also pointed out that the excess thermal energy could lead to the presence of the reaction indicated in (1). This reaction could plausibly result in surface roughening that will subsequently lead to epi-layers with morphological undulations, which will severely degrade the crystal quality of epi-layers [20].

(1) $${Sb}_{2}O_{3}+2GaSb\;\to \;{Ga}_{2}O_{3}+4Sb,$$

**Figure 2:**
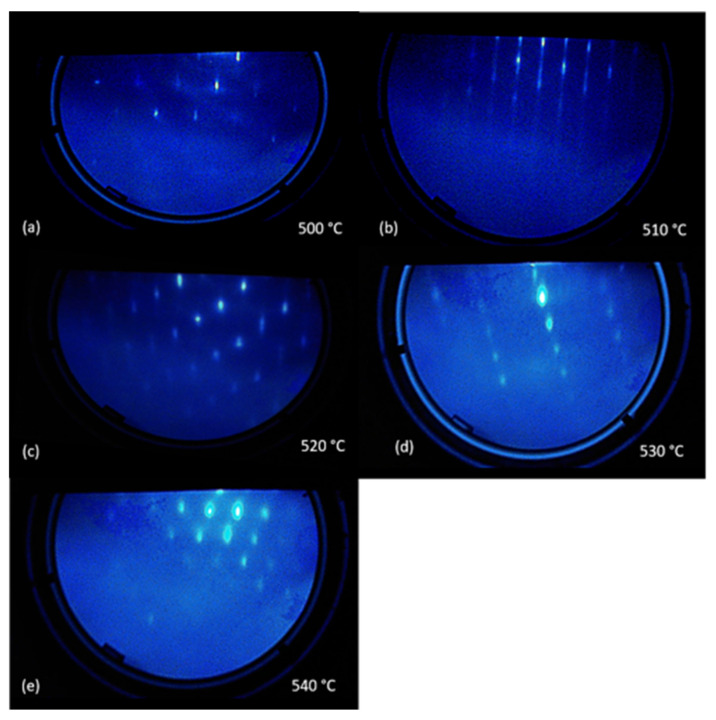
Representative RHEED patterns of the GaSb substrate surfaces after being thermally cleaned for 15 min at (**a**) 500 °C, (**b**) 510 °C, (**c**) 520 °C, (**d**) 530 °C, and (**e**) 540 °C.

To achieve a clean and smooth growth front on GaSb substrates for subsequent epitaxial growth, a systematic study was undertaken in this work by growing HgCdSe/ZnTe on GaSb with different thermal cleaning temperatures (500 °C, 510 °C, 520 °C, 530 °C, and 540 °C) while all other growth parameters and growth processes were kept the same. Figure 2 shows the in situ RHEED pattern of the GaSb substrate surface after being thermally cleaned for 15 min at the various temperatures. The spotty pattern in Figure 2a indicates the presence of oxide (non-complete thermal desorption) on the GaSb substrate surface after 15 min thermal desorption at 500 °C, while the long streak pattern in Figure 2b suggests a clean and smooth GaSb surface after 15 min thermal desorption at 510 °C. With an increase in thermal cleaning/desorption temperature to 520 °C, 530 °C, and 540 °C, a bold spotty pattern starts to appear as shown in Figure 2c–e, which indicates a rough surface potentially caused by the presence of the chemical reaction associated with (1). This in situ RHEED study indicates that thermal cleaning/desorption at 510 °C for 15 min represents the most appropriate thermal cleaning process for GaSb substrates in our MBE system.

**Figure 3:**
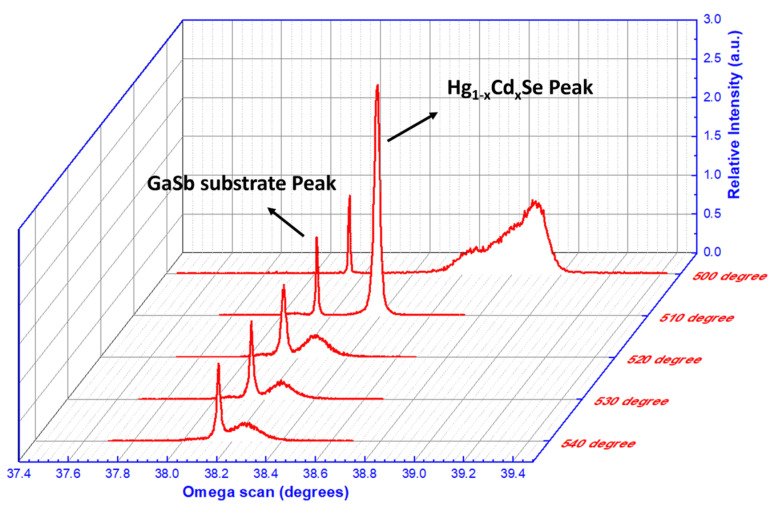
XRD rocking curves of HgCdSe/ZnTe/GaSb samples with thermal desorption at 500 °C, 510 °C, 520 °C, 530 °C, and 540 °C for 15 min. The arrows indicate the typical peaks represented by the HgCdSe active layers and GaSb substrate, respectively.

To further confirm the above RHEED study, XRD measurements were undertaken, with Figure 3 showing XRD rocking curves of the HgCdSe samples. It is evident that the HgCdSe epi-layer thermally desorbed at 510 °C presents the smallest XRD FWHM (~65 arcsec), which indicates the best crystalline quality among the HgCdSe epi-layers grown. The HgCdSe epi-layer grown on a substrate that had been thermally desorbed at 500 °C shows a very large XRD FWHM of 492 arcsec due to its non-crystalline phase (the XRD peak is not located at the same omega angle as the other samples), which is likely to be caused by the non-complete desorption of oxide from the GaSb substrate surface. However, when the thermal desorption temperature is too high, such as 520 °C, 530 °C, and 540 °C, the excess energy can lead to a rough growth front and thus epi-layers with morphological undulations that will severely degrade the crystal quality of the epi-layers, as discussed before. This results in the larger XRD FWHMs observed for the HgCdSe epi-layers grown at 520 °C, 530 °C, and 540 °C. These XRD FWHM results are well correlated with the in situ RHEED results in Figure 2 and confirm that thermal desorption at 510 °C for 15 min represents the most appropriate thermal cleaning process for GaSb substrates in our MBE system.

### 3.2. Physical Properties of HgCdSe Epi-Layers

**Figure 4:**
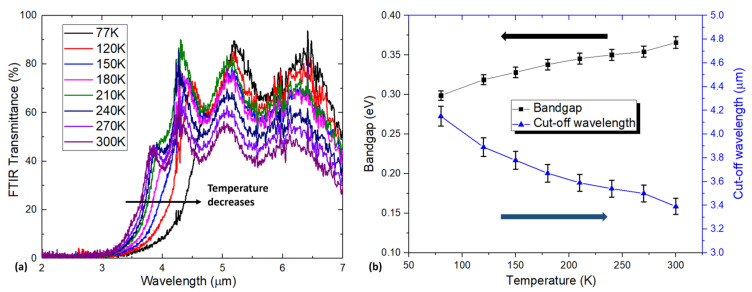
(**a**) Temperature-dependent FTIR transmission spectra of the Hg_1−x_Cd_x_Se grown with thermal desorption at 510 °C for 15 min; (**b**) temperature-dependent cut-off wavelength (blue triangle line) and corresponding bandgap (black square line) with 2% deviation tolerance.

Figure 4a presents the temperature-dependent FTIR transmission spectra of the HgCdSe sample grown on GaSb that had been thermally desorbed at 510 °C. The cut-off wavelength of this HgCdSe epi-layer shifts from ~4.2 µm to ~3.4 µm as the temperature increases from 77 K to 300 K, suggesting its potential applications for MWIR detectors. Figure 4b summarizes the temperature-dependent optical bandgap and cut-off wavelength extracted from the FTIR data in Figure 4a. As the temperature increases, the optical bandgap increases from approximately 0.30 eV to 0.37 eV, which is mainly attributed to phonon interactions and thermal expansion within the material [21]. Based on the relationship between the bandgap and the molar fraction of CdSe in the Hg_1−x_Cd_x_Se alloy (x value) developed by Summers and Broerman [9], the x value of this Hg_1−x_Cd_x_Se sample can be determined to be 0.27. Thus, this Hg_1−x_Cd_x_Se sample grown after thermal desorption of the GaSb substrate at 510 °C for 15 min will be referred to as the Hg_0.73_Cd_0.27_Se sample in subsequent discussions.

**Figure 5:**
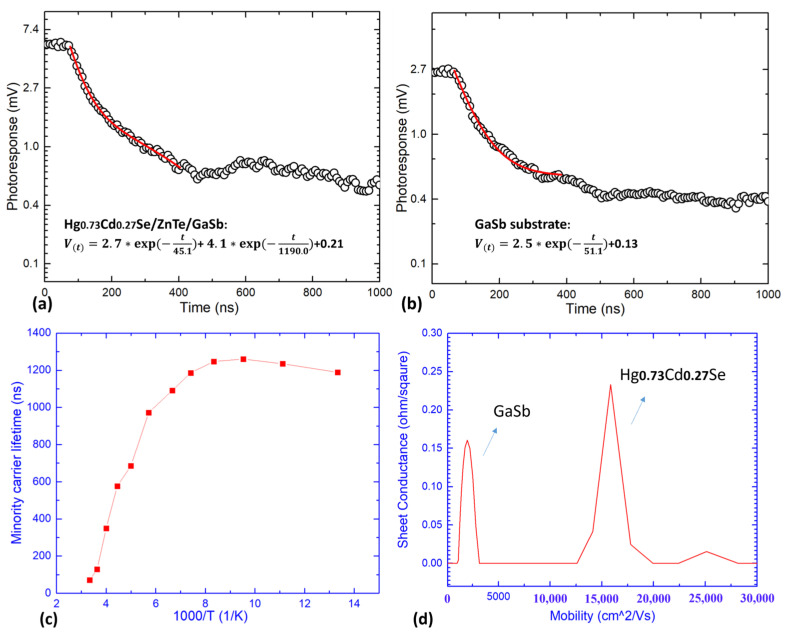
(**a**) Representative PCD measurement for Hg_0.73_Cd_0.27_Se/ZnTe/GaSb sample and (**b**) PCD measurement of GaSb (211)B substrate only at 77 K; (**c**) temperature-dependent minority carrier lifetime of Hg_0.73_Cd_0.27_Se epi-layer; (**d**) electron mobility spectrum of Hg_0.73_Cd_0.27_Se sample at 77 K.

Apart from crystalline quality and optical properties of the Hg_0.73_Cd_0.27_Se epi-layer, minority carrier lifetime and carrier mobility are the other two critical parameters which have a direct impact on detector performance. Photoconductive decay (PCD) measurements were used to measure the minority carrier lifetime in this Hg_0.73_Cd_0.27_Se epi-layer. Figure 5a shows the PCD curve of the Hg_0.73_Cd_0.27_Se sample measured at 77 K. As displayed in Figure S1, this curve is not fitted well with the commonly used single exponential function [22]; thus, a double exponential function [23] was used to fit this curve. As shown in Figure 5a, the fitted time constants are 45.1 ns and 1190.0 ns, which indicates the presence of two distinct carrier recombination mechanisms. The band gap of Te-doped GaSb substrates with a carrier concentration level of 10^17^ cm^−3^ (the GaSb substrates used in this work) is reported to be ~0.748 eV at 77 K (~1650 nm cut-off wavelength) [24]. Since the wavelength of the pulsed laser used for PCD measurements is 1550 nm, both the Hg_0.73_Cd_0.27_Se layer and GaSb substrate can contribute to the PCD signal observed in Figure 5a. To confirm and separate the signal contribution of the GaSb substrate, PCD measurements were also undertaken on a piece of GaSb (211)B substrate, the result of which is shown in Figure 5b. It is observed that the PCD curve of the GaSb substrate can be fitted well with a single exponential function with a time constant [25] of 51.1 ns, which is very close to one of the time constants (45.1 ns) extracted from the PCD curve of the Hg_0.73_Cd_0.27_Se/GaSb sample. Thus, the two distinct time constants extracted from the PCD curve of the Hg_0.73_Cd_0.27_Se/GaSb sample can be allocated to the lifetime of the GaSb substrate (45.1 ns) and the lifetime of the Hg_0.73_Cd_0.27_Se active layer (1190.0 ns). Figure 5c shows the temperature-dependent minority carrier lifetime of the Hg_0.73_Cd_0.27_Se active layer. It is observed that a microsecond-level minority carrier lifetime (>1.1 µs) can be maintained up to ~150 K, indicating the potential to achieve high operating temperatures for HgCdSe-based IR detectors.

To study the carrier transport properties, Hall measurements were undertaken on the Hg_0.73_Cd_0.27_Se sample to separate the conductivity contribution of the Hg_0.73_Cd_0.27_Se active layer from that of the GaSb substrate [26]. Figure 5d shows the HR-MSA spectrum of the Hg_0.73_Cd_0.27_Se sample measured at 77 K, which presents two distinct electron carrier species with clearly defined mobility. The electron carrier species with a concentration of ~2.9 × 10^17^ cm^−3^ and mobility of ~2.2 × 10^3^ cm^2^/Vs agree well with the parameters listed by the GaSb (211)B substrate vendor [27]. The observation of a contribution from the electron species within the GaSb substrate suggests that the ZnTe buffer layer has not completely electrically isolated the Hg_0.73_Cd_0.27_Se epi-layer from the GaSb substrate. A similar issue was also observed in previous studies as a result of the formation of electrically active defects during epitaxial growth of the buffer layer [28,29,30]. From previous research experience, the potential mitigating strategy is maintaining a low H_2_ flow during the substrate thermal desorption to achieve an atomic clean surface for the subsequent epitaxial growth [30]. The other electron carrier species with a concentration of ~2.2 × 10^17^ cm^−3^, and a mobility of ~1.6 × 10^4^ cm^2^/Vs can be assigned to the Hg_0.73_Cd_0.27_Se epi-layer. The n-type behaviour of the as-grown Hg_0.73_Cd_0.27_Se epi-layer can be attributed to the presence of native Se vacancies and other impurities [7]. As listed in Table 1, the carrier concentration of Hg_0.73_Cd_0.27_Se is around three orders of magnitude higher than typical MWIR HgCdTe, while the carrier mobility is lower by a factor of 2–3 and the minority carrier lifetime is lower by a factor of 6–7 in comparison to low-doped MWIR HgCdTe. These relatively poor carrier transport properties will inevitably degrade the device performance of the IR detectors, which will be discussed later in this work.

### 3.3. Performance Study of Prototype MWIR HgCdSe Photoconductor

**Figure 6:**
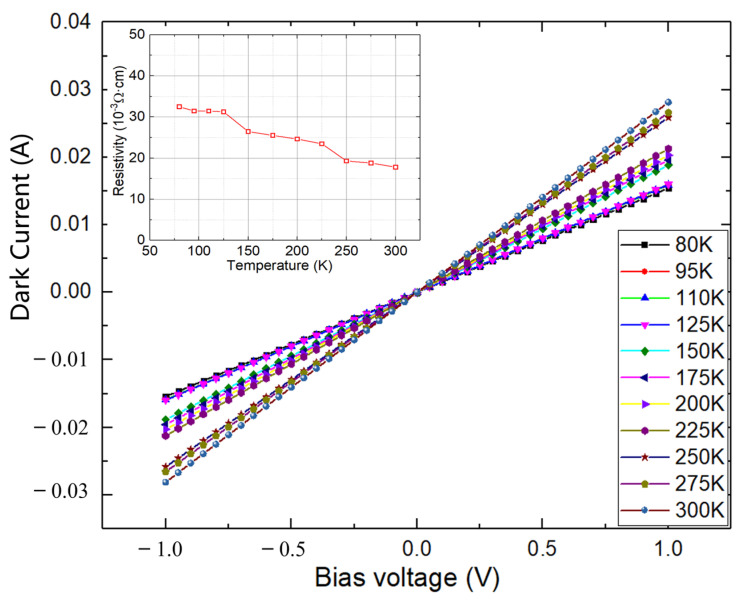
Temperature-dependent dark current–voltage characteristics of the MWIR Hg_0.73_Cd_0.27_Se photoconductive device. The inset shows the related dark resistivity at different temperatures.

**Figure 7:**
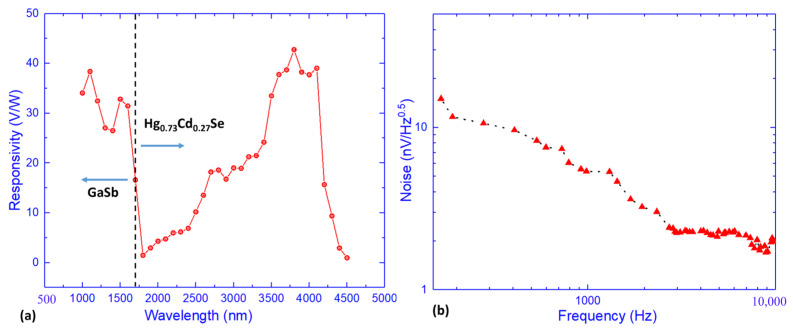
Responsivity (**a**) and noise spectra (**b**) of MWIR Hg_0.73_Cd_0.27_Se photoconductor at 77 K.

Dark current and dark resistance are important performance merits for IR detectors. Figure 6 shows the temperature-dependent dark current–voltage characteristics of this MWIR Hg_0.73_Cd_0.27_Se photoconductive device and the related dark resistance. As shown in Figure 6, the perfect linear relationship of these I–V curves indicates an ideal ohmic contact between the metal contacts and Hg_0.73_Cd_0.27_Se active layer. However, the measured dark resistance (several tens of ohms) is much lower than corresponding MWIR HgCdTe IR detectors, which are far more lightly doped and are typically around 1000 ohms. This is mainly due to the high background electron concentration (2.2 × 10^17^ cm^−3^) in the Hg_0.73_Cd_0.27_Se materials as determined by the HR-MSA analysis. This low dark resistance and thus high dark current will degrade the device performance of IR detectors, which will be discussed later in this work. To evaluate their potential for IR detector applications, MWIR photoconductor devices were fabricated based on the Hg_0.73_Cd_0.27_Se materials, and the device performance was fully characterized, including noise, responsivity, and detectivity. Figure 7a presents the 77 K spectral responsivity of the fabricated MWIR Hg_0.73_Cd_0.27_Se photoconductive detector, with the applied bias current adjusted at each temperature to accommodate the varying dark resistance and to keep the detector voltage at around 0.1 V. Thus, the influence of carrier sweepout and Joule heating can be ignored due to the low electric field (~15 V/cm) applied between the two detector electrodes [31]. Clearly, there are two photoresponse peaks observed: one cuts off at ~1650 nm, while the other cuts off at ~4200 nm. The 1650 nm cutoff is attributed to the GaSb substrate. As discussed in the previous section on carrier transport (Figure 5d, the ZnTe buffer layer does not completely isolate the conduction contribution from the GaSb substrate. Therefore, the GaSb substrate also contributes to the photoresponse signal and dominates the photoresponse signal below 1650 nm (Te-doped GaSb has an optical energy bandgap of ~0.748 eV and cut-off wavelength of ~1650 nm at 77 K). In contrast, the photoresponse peak with a cut off at 4200 nm is attributed to the Hg_0.73_Cd_0.27_Se active absorption layer, which also agrees with the cut-off wavelength measured with FTIR (see Figure 4). The MWIR Hg_0.73_Cd_0.27_Se photoconductive detector presents a peak responsivity of ~40 V/W at 77 K, which is relatively low in comparison to that of low-doped MWIR HgCdTe IR detectors (typically ~10^4^ V/W) due to the relatively high native n-type doping of the epi-layer (>10^17^ cm^−3^).

Figure 7b presents the 77 K noise voltage spectrum of the MWIR Hg_0.73_Cd_0.27_Se photoconductive detector. The 1/f noise knee point is observed around 2500 Hz, above which the noise spectrum is dominated by the g-r noise with a flat frequency response. The knee point frequency and noise spectrum characteristics observed in Figure 7b are similar to those of the MWIR HgCdTe photoconductive device reported in [31]. Based on the responsivity and measured noise spectrum, the nominalized specific detectivity ($D_{\lambda }^{*}$) can be calculated as follows:

(2) $$D_{\lambda }^{*}=\frac{R_{\lambda }}{V_{n}}{\left(lw∆f\right)}^{0.5},$$

where $R_{\lambda }$, $V_{n}$, l, w, and ∆f represent the peak responsivity, total noise voltage, width and length of device active area, and the noise bandwidth (1 Hz), respectively. Based on (2), the specific detectivity of the MWIR Hg_0.73_Cd_0.27_Se photoconductor in this work is determined to be ~1.2 × 10^9^ cmHz^1/2^/W at 77 K. This $D_{\lambda }^{*}$ value is comparable to the specific detectivity of the MWIR InAs_0.91_Sb_0.09_ photoconductor (~6.1 × 10^9^ cmHz^1/2^/W) [32] but is two orders of magnitude lower than that of low-doped MWIR HgCdTe photoconductors (~ 2.0 × 10^11^ cmHz^1/2^/W) [31].

For a typical photoconductor device, its peak responsivity can be expressed as follows:

(3) $$R_{\lambda }=\eta \frac{\lambda }{hc}er_{d}\frac{\mu_{e}E_{b}}{l}\tau_{eff},$$

where η, λ, h, c, e, $E_{b}$, l, $\mu_{e}$, and $\tau_{eff}$ represent the quantum efficiency, peak wavelength, Planck’s constant, the speed of light, fundamental electron charge, biasing electric field, length of detector (distance between two electrodes), carrier mobility, and effective carrier lifetime, respectively. In addition, $r_{d}$ represents the detector resistance, which can be expressed as follows [31]:

(4) $$r_{d}=\frac{1}{ne\mu_{e}}\frac{l}{wd},$$

where n is the thermal equilibrium electron concentration, w is the detector’s width, and *d* is the thickness of the detector active absorption layer. With (3) and (4), the relatively low device performance of the MWIR Hg_0.73_Cd_0.27_Se photoconductor compared to the counterpart MWIR HgCdTe detector can be mainly attributed to the low electron mobility, low effective carrier lifetime, and high background electron concentration as listed in Table 1. As shown by the previous study on HgCdTe materials, a low background electron concentration can significantly improve the electron mobility and effective carrier lifetime due to the reduced effects of carrier scattering, screen effect, auger recombination, and trap-assisted recombination [33]. Therefore, significant effort is required in the future to reduce the background electron concentration which will also enhance the electron mobility and the minority carrier lifetime in HgCdSe materials to improve the detector device performance. Note that this is a preliminary work, and much higher device performance is expected with more effort, such as post-growth annealing under an Se environment, using a higher-purity Se source during MBE growth, etc., which is beyond the scope of this work but provides essential topics for future study.

## 4. Conclusions

This work presents a preliminary study on the MBE growth of high-crystalline-quality HgCdSe epi-layers on GaSb (211)B substrates and the demonstration of the first prototype MBE-grown HgCdSe-based MWIR photoconductor device. It is found that thermal cleaning of the GaSb substrate surface is crucial for achieving high-crystalline-quality HgCdSe epi-layers, and the most appropriate thermal cleaning conditions are found to be 510 °C for 15 min, which leads to a record XRD FWHM (~65 arcsec) for the MWIR Hg_0.73_Cd_0.27_Se epi-layers. Although microsecond-level minority carrier lifetime has been achieved, the MBE grown Hg_0.73_Cd_0.27_Se epi-layer shows a relatively high background electron concentration which results in a relatively moderate lifetime and electron mobility, which degrades the performance of the HgCdSe-based detector. As a result, the MWIR Hg_0.73_Cd_0.27_Se photoconductor presents a cut-off wavelength of 4.2 um, peak responsivity of ~40 V/W, and peak detectivity of ~1.2 × 10^9^ cmHz^1/2^/W at 77 K. Doyle et al. proposed that applying the Se source material with higher purity and post-annealing HgCdSe epi-layers under Se overpressure condition can effectively improve the background carrier concentration by an order of magnitude [7,34]. This could be a potential research direction in our future work. Considering that this is a preliminary study, much higher device performance is expected with additional effort, which indicates the potential of HgCdSe materials for achieving next-generation IR detectors with features of lower cost and larger array format size.

## Figures and Tables

**Table 1:** Cd_0.27_Se MWIR photoconductors fabricated in this work and those of MWIR HgCdTe photoconductors reported in the open literature.

Parameters	Hg_0.73_Cd_0.27_Se (N-Type)	Hg_0.68_Cd_0.32_Te (N-Type) [31]	
Cut-off wavelength (µm)	4.2	4.5	
Minority carrier lifetime (µs)	1.19	7.90	
Mobility (cm^2^/Vs)	~16,000	~33,000	
Background carrier concentration (cm^−3^)	~ 2.2 × 10^17^	~3.4 × 10^14^	
Specific detectivity (cmHz^1/2^/W)	~ 1.2 × 10^9^	~ 2.0 × 10^11^	
	Previous Hg_1−x_Cd_x_Se research results [2]	Typical Hg_1−x_Cd_x_Te on Cd_0.96_Zn_0.04_Te substrate [1]	Hg_0.73_Cd_0.27_Se in this work
XRD FWHM (arcsec)	120–220	25–40	~65

## Data Availability

The original contributions presented in this study are included in the article/Supplementary Materials. Further inquiries can be directed to the corresponding author(s).

## Supplementary Materials

The following supporting information can be downloaded at: https://www.mdpi.com/article/10.3390/ma18153676/s1, Figure S1: Single exponential function (blue line) and double exponential function (red line) fit with PCD measurement data points for Hg_0.73_Cd_0.27_Se/ZnTe/GaSb sample.

## Abbreviations

The following abbreviations are used in this manuscript:

MBE	Molecular Beam Epitaxy
XRD	X-Ray Diffraction
FWHM	Full Width at Half Maximum
PCD	Photoconductive Decay
HR-MSA	High-Resolution Mobility Spectrum Analysis
IR	Infrared
FTIR	Fourier Transform Infrared
MWIR	Mid-Wavelength Infrared
UV	Ultraviolet
RHEED	Reflection High-Energy Electron Diffraction
