# Supplementary material for: MBE Growth of High-Quality HgCdSe for Infrared Detector Applications
Source: Materials (Basel). 2025 Aug 5;18(15):3676. doi: 10.3390/ma18153676 (PMC12348230; doi:10.3390/ma18153676)
Supplement: Supplementary file 1 [file materials-18-03676-s001.zip › materials-3727145-supplementary.pdf]

Supplementary Material for “MBE growth of high quality HgCdSe for infrared detector applications”

*Zekai Zhang, Wenwu Pan, G.A. Umana-Membreno, Shuo Ma, Lorenzo Faraone and Wen Lei**

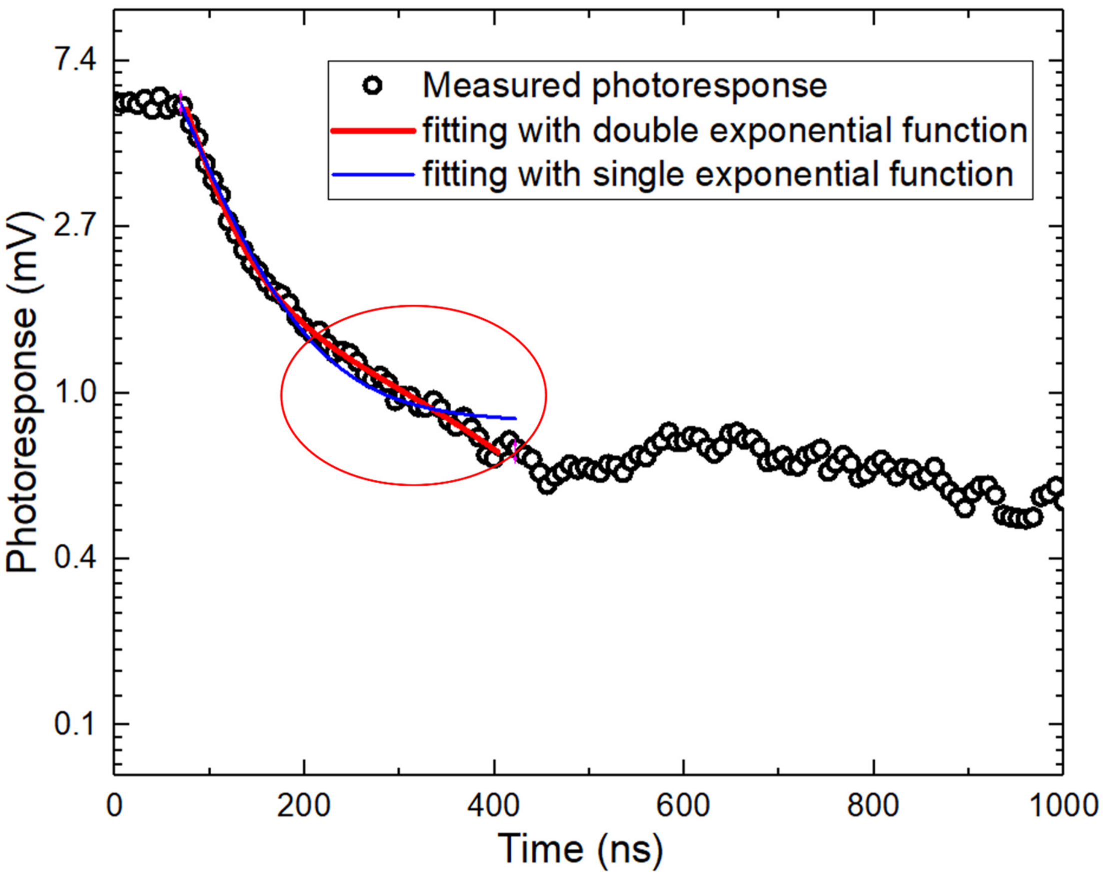

Figure S1. Single exponential function (blue line) and double exponential function (red line) fit with PCD measurement data points for Hg0.73Cd0.27Se/ZnTe/GaSb sample.
